# Phenotypes, distribution, and morphological features of antigen-presenting cells in the murine cornea following intravitreal injection

**Published:** 2007-03-28

**Authors:** Qianli Meng, Peizeng Yang, Haoli Jin, James T. Rosenbaum, Bing Li, Haining Zhang, Hongyan Zhou, Xiangkun Huang, Stephen R. Planck

**Affiliations:** 1 Zhongshan Ophthalmic Center, State Key Laboratory of Ophthalmology, Sun Yat-Sen University, Guangzhou, P.R. China; 2 Uveitis Study Center, Sun Yat-Sen University, Guangzhou, P.R. China; 3 Departments of Ophthalmology, Cell and Developmental Biology, Medicine, Casey Eye Institute, Oregon Health & Science University, Portland, OR

## Abstract

**Purpose:**

To study the phenotypes, distribution, and morphologies of different antigen-presenting cells (APCs) in the murine cornea.

**Methods:**

Intravitreal injection of fluorescently tagged ovalbumin (OVA) or antibodies to MHC-II (I-A^d^), F4/80, CD11c, B7-1, and B7-2 was performed to label cells in the murine cornea. Light and transmission electron microscopy were used to examine corneal histology. Intravital microscopy, epifluorescence microscopy, and confocal microscopy were used to evaluate the labeled cells. In vitro staining was performed to validate the in vivo staining and localize the labeled cells. Three-dimensional rotatable images were taken to evaluate relationships between two differently labeled cells.

**Results:**

Histological examination revealed no observable change in the cornea following intravitreal injection. In vivo staining showed that OVA^+^ cells and cells positive for MHC-II, F4/80, CD11c, B7-1, or B7-2 were noted throughout the cornea with a decreasing density from limbus toward the central cornea. Two populations with distinct morphological features were identified among these APCs. Labeled cells were found beneath the epithelium or in the shallow stroma in the central and paracentral cornea, but in all layers in the peripheral cornea. A number of F4/80^+^ and CD11c^+^ cells were also positive for OVA, MHC-II, B7-1, or B7-2. Rotatable images showed a close contact between two differently labeled cells.

**Conclusions:**

Intravitreal injection of labeled antibodies can be adapted to visualize labeled cells in the cornea. APCs with distinct morphologies, phenotypes, and distribution may contribute to the immunologically privileged feature of the cornea.

## Introduction

Antigen-presenting cells (APCs), such as dendritic cells (DCs), macrophages, and B cells, serve as the immune sentinels to the foreign world. DCs are characterized by expression of major histocompatibility complex (MHC) molecules, a dendritic appearance, and the capacity for presenting antigens [1-3]. They are more potent than macrophages in initiating and perpetuating secondary immune responses, and play a pivotal role in immunity and immune tolerance [4]. Macrophages are another important population of APCs. These cells are involved not only in antigen presenting processes and phagocytosis [5], but also in immune regulation in other organs and tissues due to their active secretion of a range of important biologically active molecules [6,7]. It has been shown that costimulatory molecules B7-1 and B7-2 are expressed on the surface of APCs and are involved in the activation of T cells. APCs with B7-1 mainly stimulate Th1 cells, whereas APCs with B7-2 activate Th2 cells and induce immune tolerance by producing IL-10 and IL-4 [8,9]. A recent study has shown that B7-1 and B7-2 are critical in the induction of anterior chamber-associated immune deviation (ACAID), a systemic tolerance induced by injection of soluble antigen into the anterior chamber of the eye [10]. Therefore, it seems likely that under certain conditions, B7-1 and/or B7-2 not only promote activation of T cells but also participate in the induction of immune tolerance. APCs have been found in ocular tissues such as the uveal tract [11-13], retina [14-16] and cornea [17-19]. The majority of the bone marrow (BM)-derived cells in the mouse iris-ciliary body was shown to be of macrophage and DC lineage. These APCs, particularly F4/80^+^ monocytes/macrophages, have been proposed as one of the immune regulatory components within the anterior segment of the eye that is involved in the induction of ACAID [20,21]. Moreover, as a soluble protein, ovalbumin (OVA) can be ingested, processed, and presented by professional APCs. The processing speed of OVA inside APCs is sufficiently slow to allow OVA to serve as an effective tracer reagent to study the characteristics of APCs [22].

In view of the fact that the cornea directly contacts the external environment, it is important to address the role of APCs in this tissue. Previous studies examining the cornea for APCs have largely relied on the expression of MHC-II antigens. The MHC-II^+^ cells were primarily found in the limbus and peripheral cornea of the guinea pig, hamster, mouse, and human [17-19,23-26]. However, the phenotype of these cells and their presence in the central cornea remains controversial [23,27-29]. Recent studies [30,31] identified distinct subtypes of DCs with either BM-derived DC or Langerhans cell characteristics in the murine corneal tissues. Brissette-Storkus et al. [32] have shown that the BM-derived cells that predominantly reside in the cornea stroma are macrophages. However, the phenotype, distribution, and morphological feature of APCs in the murine cornea have not been well characterized. To address these issues, the present study extensively examined murine corneal APCs by combining intravitreal injection of fluorescently tagged OVA and antibodies, intravital microscopy, whole mount ocular tissue processing, and confocal microscopy techniques. Based on the morphological features, we identified APCs of two major phenotypes throughout the cornea with a decreasing density from limbus toward the central cornea.

## Methods

### Animals

Female BALB/c mice from the animal facility at Sun Yat-Sen University (GuangZhou, China), 6-8 weeks of age and 20-26 g body weight, were used for histological examination, slit lamp microscopy, epifluorescece microscopy, and confocal fluorescence microscopy observation. A subset of studies including intravital microscopy, epifluorescece microscopy, and confocal fluorescence microscopy were also conducted on BALB/c mice with the same age and body weight housed in the animal care facilities of the Oregon Health & Science University. All experiments were performed in accordance with the ARVO Statement for the Use of Animals in Ophthalmic and Vision Research.

### Histological examination

Corneas were examined under a dissection microscope before intravitreal injection, and only those without signs of inflammation or other abnormalities were used in the studies. In order to assess if the injection technique itself altered corneal APCs, six normal BALB/c mice were anesthetized by inhalation of 1.7% isoflurane in oxygen. A glass micropipette (approximately 80 μm diameter) was fitted onto a sterile infant feeding tube and mounted onto a 0.1 ml Hamilton syringe. A Hamilton automatic dispensing apparatus was used to inject two μl isotype controls into the right eye vitreous body. The left eyes were used as controls. Twenty-four h later, the mice were sacrificed and the enucleated eyes were used for light and transmission electron microscopy. Six eyes (including 3 injected eyes and 3 normal eyes) were fixed in 4% paraformaldehyde for 24 h and used for light microcopy. Paraffin sections (5 μm) were prepared and stained with hematoxylin and eosin (H&E). The other 6 eyes were fixed in 2.5% buffered glutaraldehyde overnight and then in 1% osmium tetroxide for 1.5 h at 4 °C. The corneal tissues were then dehydrated in a graded ethanol series and embedded in Epon. Ultrathin sections were cut at 50 nm, stained in aqueous 2% uranyl acetate, and viewed under a H600 transmission electron microscope (Hitachi, Tokyo, Japan).

### In vivo immunofluorescent labeling

Intravitreal injection of fluorescently labeled reagents was performed on six mice after they were anesthetized as described above. At 1, 4, 8, 12, and 24 h after this injection, the mice were examined with a slit lamp microscope while awake. Our result showed that intensified fluorescence was observed at 1 h and thereafter. This result indicated that the intravitreously injected fluorescently labeled reagents could readily enter the anterior chamber. For the purpose of visualizing the labeled cells in the cornea, 2 μl of fluorescently tagged OVA (50 μg) or antibodies were injected into the vitreous body of BALB/c mice as described above (Table 1 and Table 2). These mice were anesthetized with one l/min of 1% to 2% isoflurane in oxygen and the labeled cells in the cornea were visualized by intravital microscopy as described previously [33] at 4, 12, and 24 h after injection. The microscopic images were captured by a Kappa CF11 DSP color video camera coupled to an image intensifier and recorded at 30 frames/s and 500x350 pixels.

**Table 1:** Groups designed for in vivo immunofluorescent staining.

**Group**	**Number of mice (eyes)**	**Reagents injected intravitreally**
1	3(6)	OVA
2	3(6)	MHC-H
3	3(6)	F4/80
4	3(6)	CDllc
5	3(6)	B7-1
6	3(6)	B7-2
7	3(6)	OVA + MHC-H
8	3(6)	OVA+F4/80
9	3(6)	OVA+CDllc
10	3(6)	MHC-H - F4/80
11	3(6)	MHC-H + CDllc
12	3(6)	F4/80 + CDllc
13	3(6)	F4/80 + B7-1
14	3(6)	F4/80 + B7-2
15	3(6)	CDllc+ B7-1
16	3(6)	CDllc+ B7-2
l7	3(6)	isotype control (mouse, hamster, rat IgG^b)

To visualize the labeled cells in the cornea, fifty-one BALB/c mice were divided into 17 groups and 2 μl of fluorescently tagged OVA or antibodies were injected into these murine vitreous bodies, respectively, according to designed combinations. Except for OVA, all were antibodies against the indicated cell surface molecules.

**Table 2:** Antibodies used in this study and their specificities.

Primary Antibody	Specificity	Companies
I-Ad	Mouse anti-mouse MHC-II antigen	Pharmingen, San Diego, CA
CDllc	Hamster anti-mouse pi50.95. DC/LC marker	Serotec, Raleigh, NC
B7-1/CD80	Hamster anti-mouse B7-1 co-stimulatory molecule	Pharmingen, San Diego, CA
B7-2/CD86	Rat anti-mouse B7-2 co-stimulatory molecule	Pharmingen, San Diego, CA
F4/80	Rat anti-mouse F4/80 monocytes-macrophages marker	Serotec, Raleigh, NC
Mouse IgG2b	Control antibody for I-Ad (MHC-II) antigen	Pharmingen, San Diego, CA
Hamster IgG	Control antibody for CDllc. B7-1	Serotec, Raleigh, NC
Rat IgG 2b	Control antibody for F4/80. B7-2	Serotec, Raleigh, NC

Different antibodies were used to examine the immune cells of the murine cornea. All primary antibodies and OVA were conjugated with either Alexa Fluor 488 (green) or Alexa Fluor 594 (red) using the Monoclonal Antibody Labeling Kit according to the manufacturer's (Molecular Probes, Eugene, OR) instructions.

The mice were sacrificed at 24 h after injection and the eyes were immediately removed and fixed in 4% paraformaldehyde in the dark for 4-6 h at 4 °C. Corneal whole-mount specimens were prepared as previously reported [34] and flattened by multiple radial cuts from the limbus to the paracentral area. The corneal whole-mounts were placed on slides with the epithelium facing upwards, embedded in Slow Fade antifade mounting medium (Molecular Probes, Eugene, OR) and covered. Standard epifluorescence microscopy with Axioplan 2 imaging and Axiophot 2 universal microscopes (Carl Zeiss, Germany) and confocal fluorescence microscopy with LSM 510 and LSM 510 META laser scanning microscopes (Carl Zeiss) were used to examine the labeled cells in the corneal whole-mounts. Three-dimensional rotatable images were generated using 64 sections from confocal microscopy with the Zeiss LSM analysis software.

Frozen sections were also made from additional mice that received intravitreal injection of the same reagents or antibodies. At 24 h after injection, the eyes were embedded in OCT and frozen immediately. Longitudinal sections with 5 μm thickness were made and viewed by epifluorescence microscopy.

### In vitro immunofluorescent staining

Corneal whole-mounts were isolated from normal BALB/c mice using the procedure described above. Corneal tissues were fixed for 30 min at 4 °C in 1% paraformaldehyde-PBS followed by extensive washing with PBS. To block nonspecific staining, corneas were incubated in PBS containing 2% bovine serum albumin for 15 min at room temperature and subsequently were blocked for 20 min at 37 °C with 10 μg/ml anti-FcγIII/II receptor monoclonal antibody (CD16/CD32; BD Biosciences Pharmingen, San Diego, CA) diluted in PBS. Afterward, corneas were immunostained with MHC-II, F4/80, or CD11c (1:40) or isotype-matched control antibodies for 2 h at room temperature. Finally, the corneas were mounted according to the method stated above and observed with epifluorescence microscopy.

### Statistical analysis

To compare the density of fluorescently labeled cells in different portions of the cornea, the cornea, roughly 3.3 mm in diameter, was divided into four parts: central, paracentral, peripheral, and limbus according to the method described previously [17]. Labeled cells were counted using a calibrated eyepiece graticule under an epifluorescence microscopy with a 20X objective lens. Counting was performed randomly in three separate fields of each unique area in the corneal whole-mounts, and the average density per square millimeter of cells was expressed as mean±SD. The randomized block design was used to compare the difference in cell density among different portions of the same cornea, and a p<0.05, as determined by the student's T-test, was considered significant. The number of eyes used for statistical analysis is shown in Table 1.

## Results

**Figure 1:**
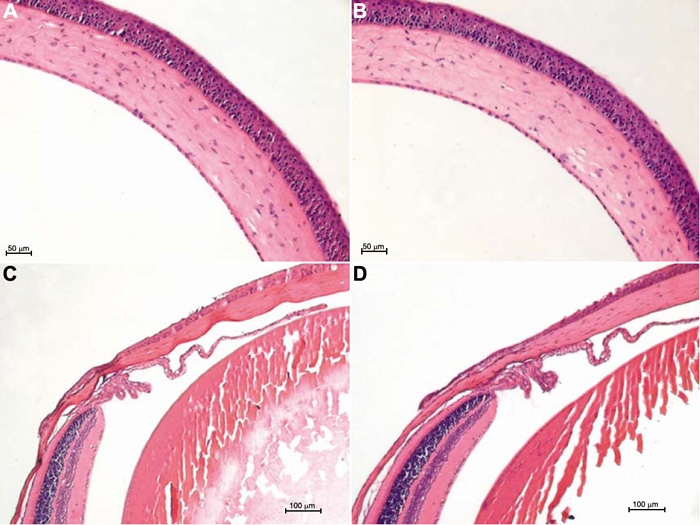
Histology of the cornea, iris-ciliary body, and retina in the normal mouse and the mouse receiving intravitreal injection. Light micrograph of the normal cornea, iris-ciliary body, and retina (**A**,**C**) and the cornea, iris-ciliary body, and retina of mice receiving intravitreal injection (**B**,**D**). The corneal epithelium, endothelium, and stroma are arranged in order and no inflammatory cells and edema are observed.

**Figure 2:**
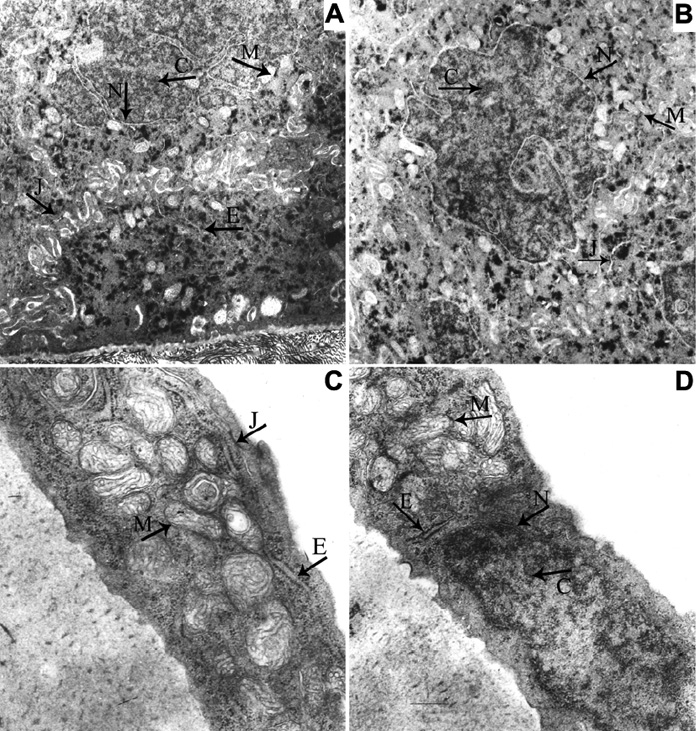
Ultrastructure of the cornea from the normal mouse and the mouse receiving intravitreal injection. Electron micrograph of the normal cornea (**A**: epithelium, **C**: endothelium) and the cornea of mouse receiving intravitreal injection (**B**: epithelium, **D**: endothelium). The mitochondrion has no ectasia, the mitochondrial outer and inner membranes are intact, and the mitochondria cristae arrange normally. The endoplasmic reticulum does not dilate. The nuclear membrane has integrity and the chromatin is distributed uniformly. The intercellular substance does not broaden and the cellular junctions were normal. The arrows indicate the location of different microorganelles. M: mitochondrion, E: endoplasmic reticulum, N: nuclear membrane; C: chromatin, J: cellular junction. The magnification in **A** and **B** is 12,000X while the magnification in **C** and **D** is 22,500X.

To examine whether intravitreal injection of 2 μl reagent could result in a visible change, we investigated the cornea using light and transmission electron microscopy. Light microscopy revealed that there was no change in the cornea, iris-ciliary body and retina from mice receiving intravitreal injection and that no inflammatory cells or edema were observed in the anterior segment of the eye (Figure 1). Transmission electron microscopy revealed a normal mitochondrial appearance and arrangement. There were no pathological changes in the cellular junction, endoplasmic reticulum, nuclear membrane, chromatin, and intercellular substance (Figure 2).

**Figure 3:**
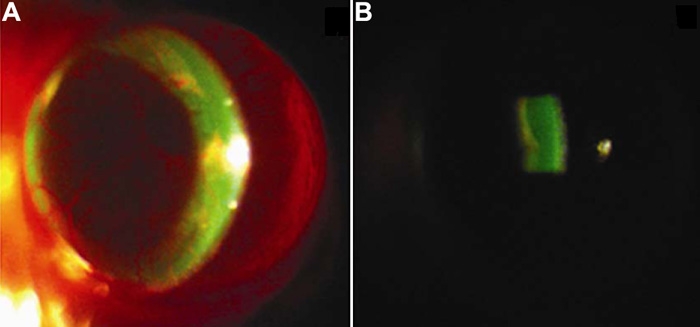
Slit lamp microscopy on the anterior segment after intravitreal injection of fluorescently labeled reagents. Intense fluorescence was observed in the anterior chamber 1 h after intravitreal injection of green fluorescently labeled OVA using slit lamp microscopy. (**A**: showing the fluorescence in the whole anterior chamber; **B**: showing the fluorescence in the central anterior chamber.)

Slit lamp microscopy was used for observing the distribution of injected fluorescently labeled reagents. Intense fluorescence was observed in the anterior chamber at 1 h (Figure 3) and was followed by a gradual decrease in the fluorescent intensity from 4 to 24 h after intravitreal injection. At 24 h after injection, the intensity of fluorescence in the anterior chamber was weak and the iris could be clearly seen. No subconjunctival accumulation of fluorescence was observed at any time point after intravitreal injection of the fluorescently labeled reagents.

**Figure 4:**
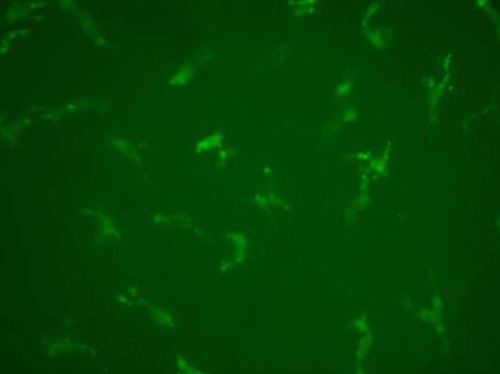
Intravital micrography of OVA+ cells in the murine cornea. OVA^+^ cells in the paracentral region of the murine cornea are detected by intravital microscopy 12 h after intravitreal injection (X200). The background is somewhat stronger.

**Figure 5:**
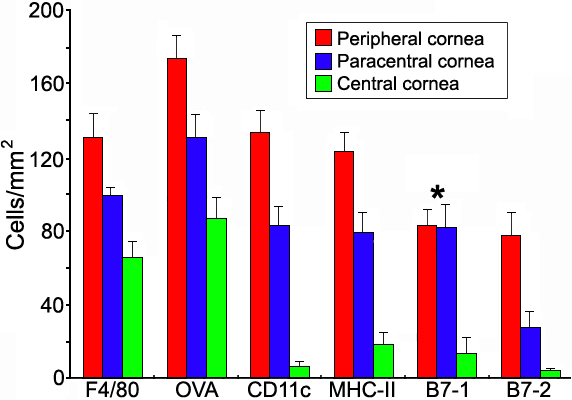
Differently labeled cells in the normal murine cornea. Density of differently labeled cells in the normal murine cornea (cells/mm^2^). A significant difference (p<0.05) in cell density among three portions (periphery, paracenter, and center) of the normal cornea is noted for all but B7-1^+^ cells (asterisk).

**Figure 6:**
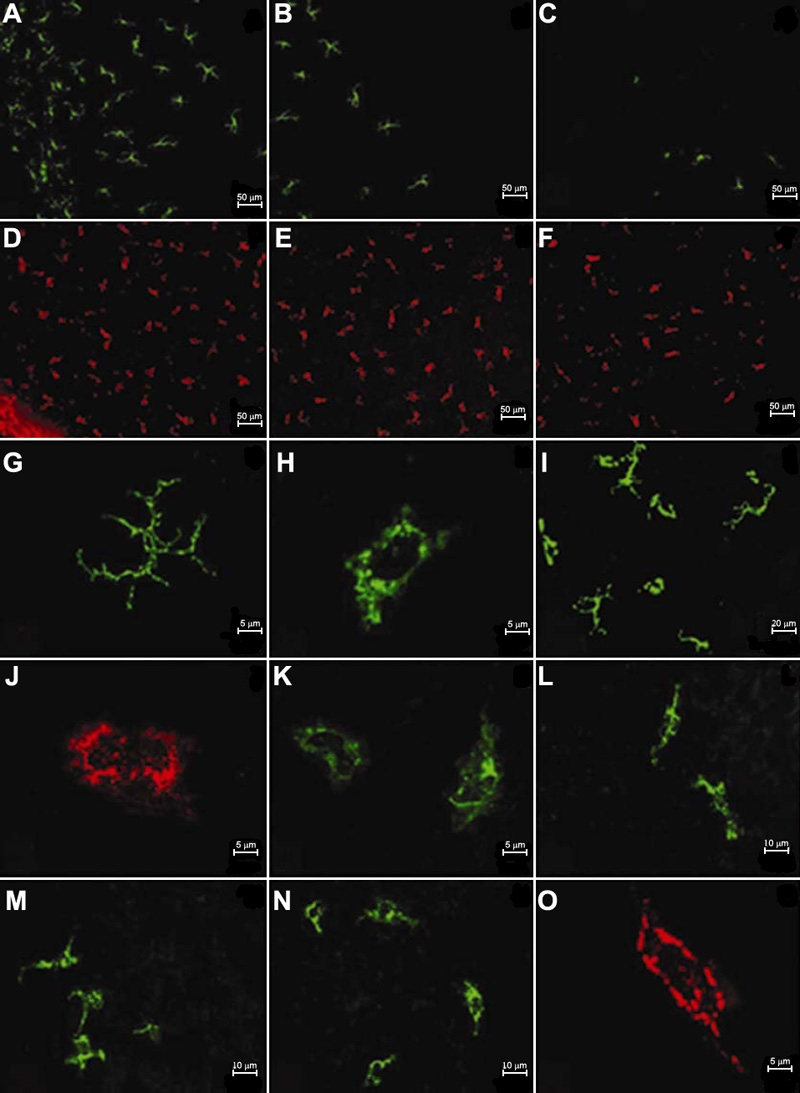
Distribution and morphology of labeled cells in the murine cornea at 24 h after intravitreal injection of fluorophore-conjugated reagents. A decreasing density of positive cells is observed from peripheral to central cornea (**A**,**D**: peripheral cornea, **B**,**E**: paracentral cornea, **C**,**F**: central cornea. **A**-**C**: MHC-II^+^ cells, **D**-**F**: OVA^+^ cells). Two populations of each antibody labeled cells, i.e., dendriform (**G**: MHC-II^+^ cell, **I**: CD11c^+^ cells) and round or irregular shapes (**H**: MHC-II^+^ cell, **I**: CD11c^+^ cells, **J**: F4/80^+^ cell, **K**: B7-1^+^ cell) are identified. F4/80^+^ cells seem to be more elongated in the peripheral area (**L**), slightly ramose in the paracentral region (**M**), and more round or oblong in the central cornea (**N**). All OVA^+^ cells show round, oblong or irregular shapes (**O**).

Cells positive for OVA, MHC-II, F4/80, CD11c, B7-1, or B7-2 were intravitally observed in different portions of the cornea by 4, 12, and 24 h after intravitreal injection. In general, the background was very strong and labeled cells were vaguely observable at 4 and 12 h (Figure 4). By 24 h, background staining was much less and labeled cells could be clearly seen. Therefore, mice were sacrificed at 24 h and the corneas were evaluated using epifluorescence and confocal microscopy. The two techniques provided similar results. There were too many labeled cells to count in the limbus. A gradually decreasing density of labeled cells was noted from limbus towards the central cornea regardless of the fluorescent-conjugated reagent used. Except for the B7-1^+^ cells, the density of different labeled cells was significantly different among peripheral, paracentral, and central cornea (p<0.05; Figure 5 and Figure 6A-F). Cells labeled with OVA usually showed somewhat stronger staining as compared with antibody-labeled cells. Staining using isotype control antibodies was rarely detected in corneal cells, therefore excluding appreciable nonspecific uptake of antibody.

As shown in Table 3, at least two morphologically different populations of cells were labeled for MHC-II, CD11c, F4/80, B7-1, or B7-2 in the murine cornea. However, morphological features were different for the each surface marker. For the MHC-II^+^ cells, approximately two thirds of them had a dendritic appearance with a small cell body, many long dendrites, and intense staining (Figure 6G). These cells were found at the limbus, peripheral, and paracentral cornea, but were undetectable in the central area (Figure 6A-C). The other one third of MHC-II^+^ cells possessed round or irregular shapes with a large cell body, a few, short dendrites, and weak staining (Figure 6H). These cells were seen throughout the cornea in a decreasing cell density from the limbus toward the center. The morphological characteristics of CD11c^+^ cells were similar to those of MHC-II^+^ cells (Figure 6I). The majority of F4/80^+^, B7-1^+^, and B7-2^+^ cells were round, oblong, or irregular, with a large cell body and a few, short dendrites (Figure 6J,K). Theses cells were dense in the peripheral area, where they were extremely elongated (Figure 6L), and also extended through the paracentral region, where they were slightly ramose (Figure 6M), and into the central cornea where they were more round or oblong (Figure 6N). Some cells were bright, while others were weakly stained. With a careful examination at medium-power magnification of 400X, we noted that a minority of F4/80^+^, B7-1^+^, and B7-2^+^ cells, similar to the first population of MHC-II^+^ cells, was also dendriform. However their staining was weaker. OVA^+^ cells, like F4/80^+^, B7-1^+^, and B7-2^+^ cells, were visualized throughout the cornea with a decreasing density from peripheral to central areas (Figure 6D-F). These cells exhibited a round, oblong, or irregular shape in the murine cornea (Figure 6O).

**Table 3:** Morphological features and distribution of positive cells in the murine cornea.

**Pheuotype**	**Morphology**	**Proportions**	**Distribution**
MHC-II+ cells or CDllc+ cells	dendritic appearance with a small cell body but many long dendrites	about two thirds	distributed at the limbus, peripheral and paracentral cornea, but undetectable in the central area
	round or irregular shapes with a large cell body and a few short dendrites	about one third	throughout the cornea in a decreasing cell density from the limbus toward the center
F4/80+ cells, B7-1+ cells or B7-2+ cells	round, oblong or irregular in appearance, with a large cell body and a few short dendrites	majority	throughout the cornea with different sliapes in different areas
	dendriform in appearance	minority	difficult to address their distribution
OVA+ cells	round, oblong or irregular shapes	all	throughout the cornea with a decreasing density from peripheral to central parts

Morphological features and distribution of labeled cells in the murine cornea at 24 h after intravitreal injection of fluorophore-conjugated reagents were observed using confocal fluorescence microscopy. The corneas were examined at medium-power magnification (400X).

**Figure 7:**
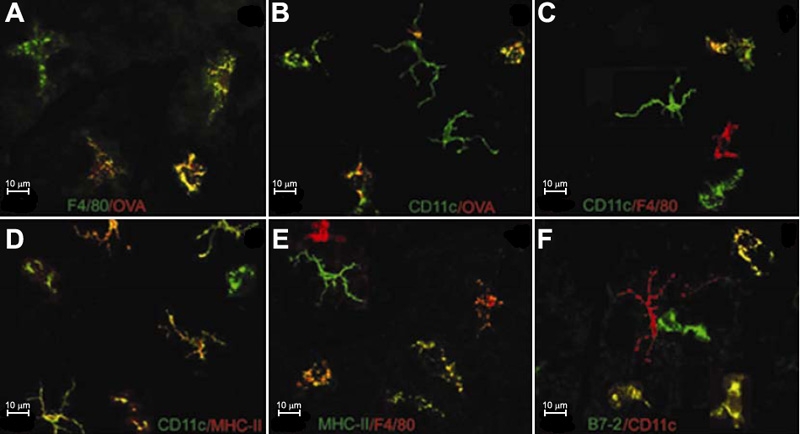
Phenotypes of APCs in the normal cornea shown in confocal microscopy images. Corneas are double stained in vivo with OVA and antibodies, followed by examination of wholemounts under the confocal microscopy. A number of F4/80^+^ (**A**: green) and CD11c^+^ (**B**: green) cells are positive for OVA (**A**,**B**: red). A few of F4/80^+^ cells are positively costained with anti-CD11c (**C**: red F4/80, green CD11c). A number of CD11c^+^ cells (**D**: green) and F4/80^+^ cells (**E**: red) are also MHC-II positive (**D**: red, **E**: green). More than half of CD11c^+^ cells are double stained for B7-2 (**F**: red CD11c, green B7-2). Double-labeled cells are yellow.

To examine the phenotypes of these positively labeled cells in the murine cornea, we costained the cornea using the antibody and OVA combinations as listed in Table 1 (Group 7 to 16). About 90% of F4/80^+^ and 57% of CD11c^+^ cells, mostly showing round or irregular shapes, were positive for OVA (Figure 7A,B). Only about 25% of F4/80^+^ cells were positively costained with anti-CD11c (Figure 7C). Approximately 91% of CD11c^+^ cells and 69% of F4/80^+^ cells were MHC-II positive (Figure 7D,E). Approximately 60% of CD11c^+^ cells (Figure 7F) and 50% of F4/80^+^ cells were B7-1 or B7-2 positive. About 52% of OVA^+^ cells were MHC-II^+^ cells with round or irregular appearance (Table 4). There was no difference between peripheral and central cornea regarding the coexpressive ratios of cell surface markers.

**Table 4:** Coexpression of cell surface markers on normal murine corneal cells.

**Marker one**	**Marker two**	**Cells expressing marker one that coexpress marker two (%)**
F4/80	OVA	89.7 ± 8.9%
F4/80	CDllc	24.8 ±9.1%
F4/80	MHC-II	68.7 ± 10.7%
F4/80	B7-1	51.6 ± 11.4%
F4/80	B7-2	53.8 ± 16.3%
CDllc	OVA	56.9 ± 12.6%
CDllc	MHC-II	91.4 ±7.3%
CDllc	B7-1	58.3 ±17.4%
CDllc	B7-2	62.7 ± 15.2%
OVA	MHC-II	52.0 ± 15.2%

To examine the phenotypes of these positively labeled cells in the murine cornea, the cornea were costained with the combinations of antibodies and OVA in two-color phenotype analysis.

**Figure 8:**
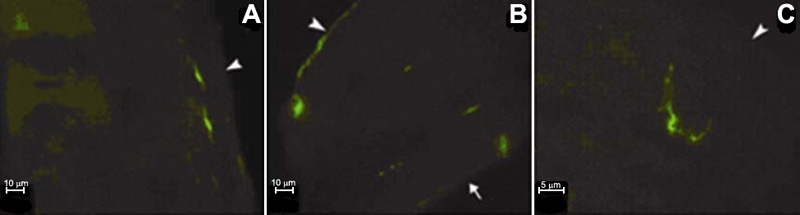
Location of labeled cells in the vertical axis of the cornea. Frozen sections are prepared and stained with antibodies and OVA, followed by epifluorescence microscopy examination. OVA^+^ cells are distributed beneath the epithelium in the central part (**A**) and present in all layers of the peripheral cornea (**B**). The dendrites projecting from MHC-II^+^ cells are present longitudinally into the epithelium and stroma (**C**). Arrowheads indicate the epithelium location and the arrow indicates the location of endothelium.

To assess the locations of the positively labeled cells in the anterior-posterior axis of the murine cornea, frozen sections were prepared following in vivo immunofluorescent staining. Cells positive for OVA and F4/80 were detected beneath the epithelium or in the anterior stroma and paralleled the arrangement of corneal collagen fibrous sheets in the central and paracentral cornea. However, these cells were present in all layers of the cornea at the peripheral part (Figure 8A,B). The locations of MHC-II^+^ and CD11c^+^ cells were similar to those of OVA^+^ and F4/80^+^ cells, except that dendrites projecting longitudinally into the epithelium or into the stroma were occasionally seen (Figure 8C).

**Figure 9:**
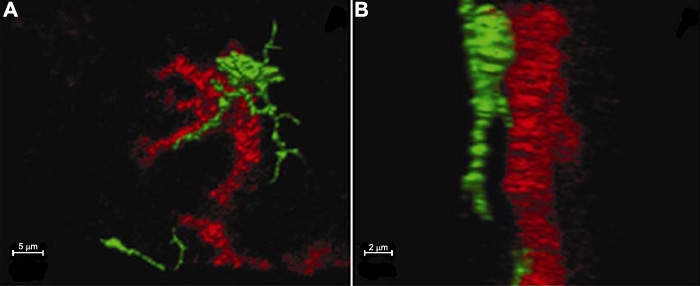
Three-dimensional rotatable confocal microscopy images for observing the relationship between labeled cells. The MHC-II^+^ cell contacts an OVA^+^ cell at a portion of the cell body and the dendrite (**A**: the frontal image, **B**: the lateral image; red OVA, green MHC-II).

Three-dimensional rotatable images disclosed a close relationship between certain labeled cells. Interestingly, a few cells seemed to contact each other at a portion of the cell body or the dendrites (Figure 9), although the physiological role of these contacts remains to be investigated.

**Figure 10:**
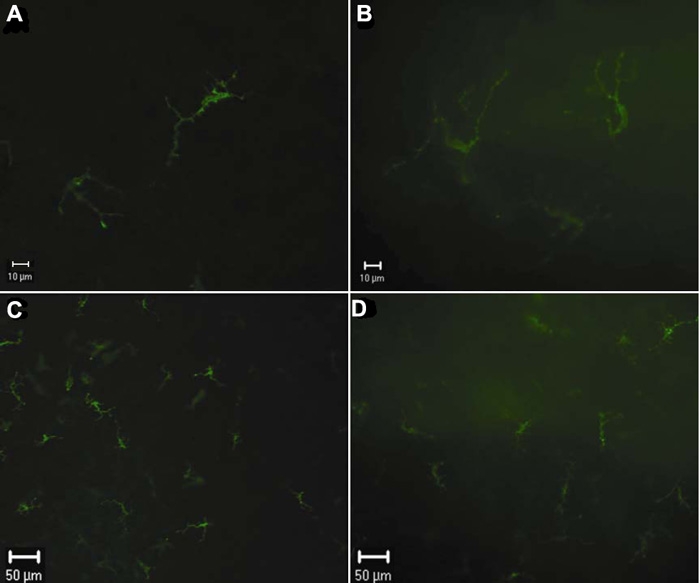
MHC-II positive cells stained in vivo and in vitro shown by epifluorescence microscopy. MHC-II^+^ cells stained in vivo 24 h after intravitreal injection (**A** and **C**) and in vitro (**B** and **D**) in the paracentral region of the murine cornea are observed by epifluorescence microscopy. The background in vitro staining is stronger than that in vivo staining. The labeled cells in vitro staining appear to be vaguely seen as compared with those in vivo staining.

To validate the in vivo staining experiments and eliminate the possibility that cells were labeled because of nonspecific antibody ingestion, we performed the in vitro immunofluorescent staining using antibodies to MHC-II, F4/80 and CD11c. Cells identified in the cornea were comparable with those observed by in vivo staining. However, the in vitro staining resulted in a stronger background and the labeled cells were vaguely observed (Figure 10).

## Discussion

Using confocal fluorescence microscopy and other techniques after intravitreal injection of fluorescently tagged antibodies, we have demonstrated that there are at least two populations of APCs with a decreasing density from limbus towards center in the murine cornea. All the positively labeled cells were found beneath the epithelium or in the anterior stroma in the central and paracentral cornea but were noted in all layers of the peripheral cornea. Double staining revealed that a number of F4/80^+^ and CD11c^+^ cells were also positive for OVA, MHC-II, B7-1, or B7-2. Rotatable images showed a close contact between certain labeled cells.

Previously we used an intravital microscopy technique to visualize cells in the iris labeled via injecting relevant reagents or antibodies into the anterior chamber [35]. However, this route of injection could damage the cornea despite cautions taken during the injection. In this study, we developed a useful method of labeling cells in the cornea by intravitreal injection of fluorescently tagged reagents coupled with intravital microscopy and confocal fluorescence microscopy. Using these techniques, our study showed that the injected reagents readily enter the anterior chamber, as evidenced by intense fluorescence in the anterior chamber shortly after intravitreal injection. Although a barrier was generally thought to be present at the endothelial level of the cornea, these fluorescently labeled reagents could subsequently penetrate into the cornea with negligible damage to the eye. As a result, labeled cells could be clearly observed using confocal microscopy and there was only weak background in the corneal whole-mounts at 24 h. In order to compare the similarity between in vivo staining and in vitro staining, two complementary experiments, i.e., in vitro staining and isotype-matched control antibody staining were performed to exclude the artifactual effect and validate results of the in vivo staining. In vitro immunofluorescent staining of corneal wholemounts also demonstrated that the positively labeled cells were not the result of nonspecific ingestion of antibodies. Cell morphologies and distributions acquired from these in vitro staining results were essentially identical to those of in vivo staining, indicating the de facto presence of these cells in the cornea. Moreover, the control experiment using isotype-matched immunoglobulin excluded the possibility of nonspecific staining.

Our study showed that intravitreal injection of fluorescently labeled antibodies did not influence the cornea histologically or ultrastructurally. In conventional histological or immunohistochemical studies, one cannot eliminate the possibility that death and/or fixation alter the detection of cell surface molecules. Comparing fixed tissue staining with staining of live tissue, we believe that background staining is minimized in vivo. In fact, this technique omits a number of procedures and reagents used in in vitro experiments, thereby enhancing the reliability of the results. Moreover, intravitreal injection of fluorescently labeled antibodies may allow a dynamic observation on the labeled cells in the cornea using intravital microscopy. Therefore, combination of intravitreal injection of labeled reagents with intravital microscopy may be a useful technique to follow changes of immune cells in the cornea.

APCs in the cornea have received enormous attention during the past decades, mainly because of the concept that the cornea is an immunologically privileged tissue. Our studies confirm the previously described results in which APCs are distributed more densely in the limbus and peripheral cornea than in the paracentral or central cornea [17]. Numerous APCs are present in the limbus and in the portion of the cornea adjacent to it. The densely distributed APCs in the limbus and peripheral cornea are of great importance to the immune surveillance in the cornea and may also be pathologically involved in the development of inflammatory diseases frequently seen in the peripheral cornea [36]. Frozen sections revealed that all of the positive cells were located beneath the epithelium or in the anterior stroma and paralleled the arrangement of corneal collagen fibers in the central and paracentral cornea. Three-dimensional images obtained using confocal microscopy disclosed a flat-shaped morphology of the labeled cells in the central or paracentral cornea, with a few dendrites of MHC-II^+^ and CD11c^+^ cells projecting longitudinally into the corneal epithelium or stroma. The morphological features and arrangement of these APCs in the cornea may not only allow the light to readily enter the eye but also contribute to the corneal transparency.

Our studies revealed that a number of OVA^+^ cells were positive for CD11c and F4/80, suggesting that these cells might be able to take up and process foreign proteins and probably belonged to the macrophage and DC lineages. Most F4/80^+^ cells were positively stained for OVA, indicating that they probably had a strong ability to phagocytose or pinocytose antigens. About a quarter of these F4/80^+^ cells might be DCs because they were positive for CD11c. These results are consistent with the notion that the monoclonal antibody to F4/80 recognizes both macrophages and DCs [37]. Almost all CD11c^+^ cells were MHC-II positive, suggesting that the phenotype of primary antigen-presenting cells in the cornea was CD11c^+^MHC-II^+^. Moreover, we found that a subset of F4/80^+^ or CD11c^+^ cells in the central and peripheral cornea were positive for either B7-1 or B7-2. It is worthwhile to mention that the cornea contains different subsets of APCs with distinct morphological features and that these APCs are partially CD11c and F4/80 positive [30-32,38]. We detected cells expressing B7-1 and B7-2 in the central cornea more readily than in one report [31] and we detected CD11c more readily than was noted in vitro [32]. The discrepancies of these results may lie in the techniques employed. Our in vivo labeling technique may allow the antibodies to penetrate into the cornea more readily than did the antibodies in in vitro experiments. The combination of in vivo staining with confocal microscopy of high resolution and sensitivity provides more precise evaluation on labeled cells in the cornea.

APCs have been suggested to play a pivotal role in initiating an immune response or inducing an immune tolerance within the cornea [39]. Using three-dimensional rotatable imaging, our study showed that a few positive cells appeared to contact each other, suggesting a cell-cell communication between certain cells. Although we do not know how these cells interact and whether the contact between them is specific or nonspecific, this direct intercellular contact may have important implications, both physiologically and pathologically.

In conclusion, we have developed a technique to study the morphologies and phenotypes of corneal cells by injecting fluorescently tagged antibodies into the vitreous body. Our study confirms the heterogeneity of corneal APCs and includes three novel observations. First, we have shown that these APCs can be identified using immunohistology in vivo. This technology will allow cell populations to be tracked in living animals. Second, we have shown that antibodies that are injected intravitreally readily enter the murine cornea. Third, we have shown a surprising juxtaposition of APCs within the cornea, a physical contact that may facilitate biological important interactions. Together with other factors in the ocular microenvironment, APCs with distinct morphologies, phenotypes and distribution in the cornea regulate the immune response and maintenance of the immunological privilege of the cornea.
